# Decreased mandibular cortical bone quality after botulinum toxin injections in masticatory muscles in female adults

**DOI:** 10.1038/s41598-020-60554-w

**Published:** 2020-02-27

**Authors:** Seok Woo Hong, Jeong-Hyun Kang

**Affiliations:** 1 Department of Orthopedic Surgery, Kangbuk Samsung Hospital, Sungkyunkwan University School of Medicine, 29, Saemunan-ro, Jongno-gu Seoul, 03181 Korea; 2 Clinic of Oral Medicine and Orofacial Pain, Institute of Oral Health Science, Ajou University School of Medicine, 164, Worldcup-ro, Yeongtong-gu, Suwon, Gyeonggi-do 16499 Korea

**Keywords:** Osteoporosis, Osteoporosis

## Abstract

This study aimed to clarify how masticatory muscle atrophy induced by botulinum toxin (BTX) injection affects cortical bone quality of the mandible using 3D modeling technology. A total of 39 young (26.9 ± 6.0 years) and 38 post-menopausal (55.3 ± 6.3 years) females were included. Computed tomography (CT) images were obtained before and after 12 months of treatment. Predictor variables were application of a stabilization splint, and/or two times of BTX injection in the bilateral temporalis and masseter muscles within a six-month interval. Outcome variables were changes in average Hounsfield units (HU) and cortical thickness of region of interest (ROI). 3D mandibular models were reconstructed using CT images, and models were used to calculate average HU and cortical thickness of ROIs, including inferior half of the lateral surface of ascending ramus, coronoid process, and temporomandibular joint condyle. Cortical bone quality at muscle insertion site was influenced by decreased muscle thickness but seemed not to be affected by decreased functional loading. Reduced functional loading seemed to influence cortical bone quality of the condyles. These effects were more remarkable in post-menopausal females. Hence, decreased masticatory muscle thickness may lead to alterations of the mandibular cortical structures, especially in post-menopausal females.

## Introduction

Bone is a dynamic structure that continuously undergoes a remodeling process through resorption and apposition to meet environmental requirements. The adaptation rate of the bone to functional loading is mediated by dynamic forces of the associated muscles^1^. Skeletal muscles transduce mechanical loading to the bone in three ways: (1) the tensile force developed by a muscle contraction at its insertion site, (2) the compressive force developed by muscles that transduce loading across the joints, and (3) the bending force experienced by long bone associated muscles for lifting distally held objects^2^. The role of mechanical stimulation by muscles to maintain bone density has been well known and the association among the amount of physical activity, body muscle mass, and quality of both cortical and trabecular bones has also been investigated^3–6^.

Botulinum toxin (BTX) acts as a pre-synaptic neurotoxin that blocks neuromuscular transmission by inhibiting acetylcholine release from the motor and sympathetic nerve terminals^7^ as well as inflammatory mediators including substance P and glutamate^7,8^. Muscle paralysis induced by BTX injection was related with not only reduced active loading but also an increased passive elastic modulus of muscle fiber bundles^9^. BTX has been used not only for cosmetic purposes but also to improve symptoms associated with temporomandibular disorders (TMDs), myofascial pain syndrome, and headaches. The majorities of adverse effects associated with the application of BTX in the orofacial region are mild and transient including bruising, swelling, pain around the injection site, and masticatory muscle weakness^10^. Several previous studies have revealed the therapeutic effects of BTX injection in masticatory muscles for the management of myofascial TMD^11–14^. Furthermore, another study reported that repeated injection of BTX would be helpful for maintaining a decreased biting force^15^.

BTX-induced muscle atrophy and paralysis could represent one of the most commonly explored animal models that can be used in the bone metabolism research. Several animal studies have attempted to show that muscle paralysis by intramuscular injection of BTX in the hindlimbs and masticatory muscle atrophy induced by BTX injection could produce bone loss^6,16–25^. Interestingly, one animal study using rodents showed that bone loss induced by intramuscular BTX injection seemed to be not solely the result of reduced mechanical loading, but bone loss could be directly affected by atrophic muscle itself^25^. The majorities of studies which tried to identify the mechanism of bone-muscle interactions using intramuscular BTX injection were performed on animal, and only a few human studies have been carried out to elucidate this phenomenon.

Craniofacial bones, especially the mandible, are not weight bearing part of the skeleton, and the main mechanical stimulation in this area is generated not from gravitational loading but by masticatory performance. When the elevator muscles, including the masseter and temporalis muscles are functioning, force is applied to the two temporomandibular joint (TMJ) condyles and tooth bearing alveolar bone^26^. According to animal studies using rodents, reduced masticatory muscle thickness resulting from BTX injections in the temporalis and/or masseter muscles could lead to changes in cortical and trabecular bone structures in masticatory load-bearing areas, the TMJ condyles and alveolar bones^17,21,23,24^. Other animal studies using rabbits showed similar results^16,18^. Most studies that investigated the relationship between BTX-induced masticatory muscle atrophy and mandibular bone loss were animal studies, and only a few human studies have performed a comprehensive analysis of bone quality and muscle thickness. One study demonstrated reduced condylar trabecular bone density in patients with TMDs who received BTX treatment in their masticatory muscles^27^. However, this study used a subjective method to evaluate changes in the trabecular density, therefore, the reliability of the results was inevitably compromised. Another study showed cortical thinning at the anterior portion of the condyles and decreased bone textures in TMJ condyles and digastric fossa, but this study could not provide information on changes in the cortical bone qualities in muscle insertion sites and adopted two-dimensional (2D) method to evaluating the cortical bone qualities^28^.

Cortical bone thickness and density are some of the main parameters that can be used to evaluate the stability of the bone. Peripheral quantitative computed tomography (pQCT) and micro computed tomography (CT) could provide sufficient information on the distribution of bone density in a region of interest (ROI), including both cortical and trabecular bone. However, the utility of pQCT is questionable in routine clinics due to its high cost, and the application of micro CT to human subjects is quite impossible. The capabilities of clinical CT to characterize structural properties of human skeletons have been explored. Predicting cortical bone densities and thickness by cortical shells of the ROIs using clinical CT provided excellent correlations with micro CT results^29,30^. The accuracy of bone densities calculated from Hounsfield units (HUs) from CT data has been demonstrated^31^, and three-dimensionally (3D) reconstructed axial CT images could accurately define the border of the cortical bone^32,33^. Therefore, owing to advances of 3D-modeling technology using CT images, cortical bone density and thickness of craniofacial structures could be evaluated in routine clinics.

To the best of our knowledge, few study has ever investigated the effects of BTX injection in masticatory muscles on the cortical bone properties in human craniofacial structures in the 3D manners. Several preclinical studies demonstrated decreased masticatory loading and tensile strength induced by intramuscular BTX injection in masticatory muscles may be associated with cortical bone quality in the craniofacial area, however sparse human studies using 3D modeling technologies have ever attempted to bring this to light. We hypothesized that the decreased masticatory muscle thickness and/or decreased functional loading would elicit decreased cortical bone quality of the masticatory load bearing area and masticatory muscle insertion sites of the mandible. Therefore, the aim of the present study was to clarify the effects of the masticatory muscle atrophy induced by BTX injection and decreased masticatory functional loading on the cortical bone quality in the mandibular structures using 3D modeling technology.

## Materials and Methods

### Participants

The present study was a retrospective cohort study using the clinical and radiographic data of TMD patients who visited the TMJ·Orofacial Pain Clinic in a tertiary University Medical Center from March, 2017 to February, 2019. Patients with the following conditions were excluded from the study: autoimmune diseases, including rheumatoid arthritis, lupus erythematosus, and fibromyalgia; craniofacial anomalies; endocrinological disorders that could affect bony metabolism, such as thyroid disease, adrenal failure, and kidney diseases; history of head and neck trauma; degenerative bony changes on either side of the TMJs; wearing removable dentures; history of having BTX injection in the masticatory muscles; a history of orthodontic treatment, lower face reshaping, or orthognathic surgery; and a history of taking bisphosphonate and/or hormone modulating agents.

Patients were diagnosed based on the Diagnostic Criteria for TMD (DC/TMD) Axis I^34^, and patients presenting with myofascial pain and myofascial pain with referral were included. All patients who received CT scans, due to click/crepitus history during examinations were included; however, no pathologic bony change was observed on CT. CT images were obtained before treatment (T0) and after 12 months of treatment (T1). In all patients, no arthritic condylar bony changes were detected during the treatment period.

The imaging data from a total of 39 young females and 38 post-menopausal females who exhibited click/crepitus during clinical examinations but did not show pathological bony changes on CT images were included in the present study. Patients were classified into four groups. Among the 77 patients, 12 young females and 10 post-menopausal females were treated only with stabilization splint (SS) therapy for 12 months (SP) to reduce masticatory loading to the TMJ condyles, 11 young females and 12 post-menopausal females were receive two sets of bilateral BTX injection in the temporalis and masseter muscles (BTXINJ) with six-month interval, 10 young females and 10 post-menopausal females were receive both two sets of BTX injection in their temporalis and masseter muscles with six-month interval and underwent SS therapy for 12 months (SPBTX), and 6 young females and 6 post-menopausal females were treated with only habit controlling treatment and had routine check-up but decided not to be treated with either SS therapy or BTX injection (CON) for 12 months. Even though, wearing a SS and/or BTX injection in the temporalis and/or masseter muscles would be beneficial for managing myofascial pain in the orofacial area, some patients did not agree to perform it due to economic problems and other reasons. One orofacial pain and TMD specialist (JHK) performed interview and provided several options to the patients including physical therapy, SS therapy, and BTX injection and final decisions were made by patients. Patients in CON were also obtained routine check-up and CT taking after 12 months of treatment because they also exhibited crepitus or click at initial examination.

The research protocol was reviewed in compliance with the Helsinki Declaration and approved by the Institutional Review Board of the Ajou University Hospital (AJIRB-MED-MDB-19-024). The Institutional Review Board committee approved a request to waive the documentation of informed consent due to the retrospective design of the study.

### Diagnosis of TMD and evaluation of the trigger points (TrPs)

The DC/TMD Axis I criteria was applied to classify myofascial TMD, and patients who were diagnosed as myofascial pain or myofascial pain with referral were included. One TMD and orofacial pain specialist (JHK) was responsible for assessing TMD and TrPs. Clinical parameters such as the extents of comfortable mouth opening (CMO) without pain and maximum mouth opening (MMO), and duration of the orofacial pain were examined. CMO and MMO measurements from the distance between the incisal edge of the central upper incisor of the right side and the same point of the lower incisor were obtained. A visual analog scale (VAS) was used to determine the severity of myofascial pain. Myofascial TrPs were bilaterally evaluated in the temporalis and masseter muscles. TrPs were evaluated on the basis of the criteria suggested by Simon and Travell^35^.

### BTX injection

Because the present study included myofascial TMD patients, a BTX injection protocol was followed for the orofacial pain management which was adopted by previous reports^14,36^. BTX in the form of a freeze-dried powder (Onabotulinumtoxin A; BOTOX^®^, Allergan Inc., Irvine, CA, USA) was reconstituted at concentration of 20 units/mL (2 units/0.1 mL, 100 units in 5 mL of sterile saline), and used immediately after preparation. The BTX preparation was injected into each temporalis and superficial masseter muscle at a dose of 20 units and 25 units per muscle, respectively. Patients in BTXINJ and SPBTX received two sets of BTX injections, with the first injection being administered at T0 and the second one being administered at 6 months after T0 and six months before T1.

### Intervention for the masticatory loading with stabilization splint (SS) therapy

SS therapy has been regarded as a frequently used tool for the treatment of TMDs. Even though the specific mechanism of SS treatment has been still controversial, one of the most widely accepted arguments was that it reduces abnormal masticatory muscle activity and mechanical loading to associated muscles and condyles, finally bringing neuromuscular balance and stability^37^. SS therapy could cause patients with TMD to reduce or stop parafunctional habits, removal of occlusal interference, reducing the masticatory loading to the TMJ condyles and teeth bearing alveolar bone, and finally resulting in reduction of the orofacial pain^38,39^. The loading from masticatory activities to the TMJ condyles comes from functional activities, such as chewing, speaking, and swallowing as well as parafunctional activities, including nocturnal clenching or grinding the teeth^26^. It has been estimated that the masticatory muscle activities from parafunctional activities are over 3 times greater than those from functional activities per day and parafunctional activities which occur in eccentric positions may lead to translation of condyles far from their stable positions^26^. Hence, controlling parafunctional activities during night would be very important for managing the orofacial pain, masticatory loading, and muscle tensions. Previous studies suggested that SS therapy relieved excessive loading on the mandibular glenoid fossa and TMJ condyles and influenced the adaptive remodeling potential in the TMJ associated structures^40,41^.

**Figure 1:**
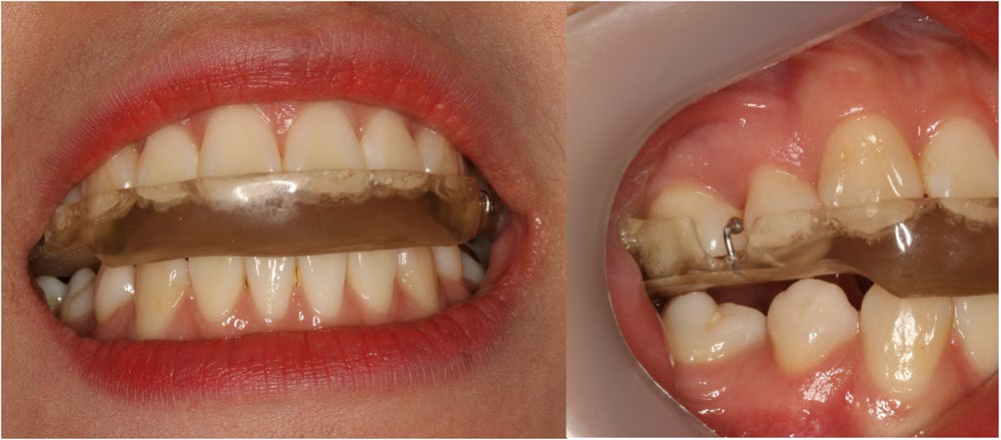
Intraoral photo of stabilization splint (SS) (**a**) Frontal view (**b**) Lateral view.

The SS was fabricated in acrylic resin with 2 mm thickness at the molar area and it covered all of the maxillary teeth. Occlusion of the SS with the lower teeth was provided to create uniform points of contact for the lower functional cusps against the SS on occluding premolar and molar teeth (Fig. 1). Patients in SP and SPBTX were instructed to wear the SS every night for at least eight hours per day during treatment period. We regarded continuous wearing of SS more than eight hours per day as valid wearing and considered as compliant with SS therapy. All participants visited the clinic monthly for routine check-ups and confirmation of compliance. The average compliance rate was 85.7% and 92.4% in SP and SPBTX, respectively.

### Measurement of muscle thickness

**Figure 2:**
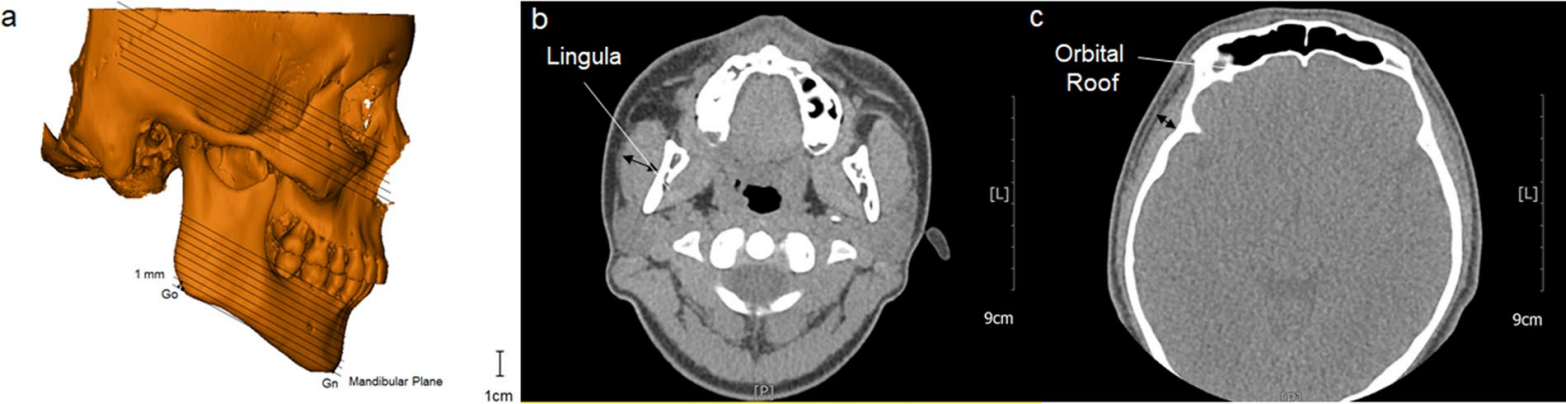
(**a**) The TMJ CT images were digitally cut into 1 mm thick sections that were parallel to the mandibular plane. (**b**) The maximum thickness of the masseter muscles were estimated on the sectioning plane, which passes through the lingula (**c**) The maximum thickness of the temporalis muscles were estimated on the sectioning plane, which passes through the orbital roof.

The TMJ CT images were digitally cut into 1 mm thick sections that were parallel to the mandibular plane. The maximum thickness of the temporalis and masseter muscles were estimated on the sectioning plane, which passes through the orbital roof and lingula, respectively (Fig. 2).

### Reconstruction of the 3D mandibular model

Previous reports that dealt with changes in pathologic TMJ condylar bony changes after conservative treatment adopted a twelve-month interval of CT taking^40–42^. CT images were obtained before treatment (T0) and after 12 months of treatment (T1). All the CT images were acquired with the same device, using a 16-section multidetector row CT scanner (SOMATOM Sensation 16, Siemens Medical Solutions, Erlangen, Germany) and 64-section multidetector CT scanner (Brilliance 64, Philips Medical System, Netherland). The CT scanning protocol was used as follows: 120 kVp tube potential; 250 mAs tube current-time product; 300 mm × 2000 mm display field of view; maximum scanning time 10 seconds; pixel size 0.3 × 0.3 mm; and 1 mm section thickness. Corrected sagittal, coronal, and axial images of the both condyles were exported into Digital Imaging and Communications in Medicine (DICOM) file format.

**Figure 3:**
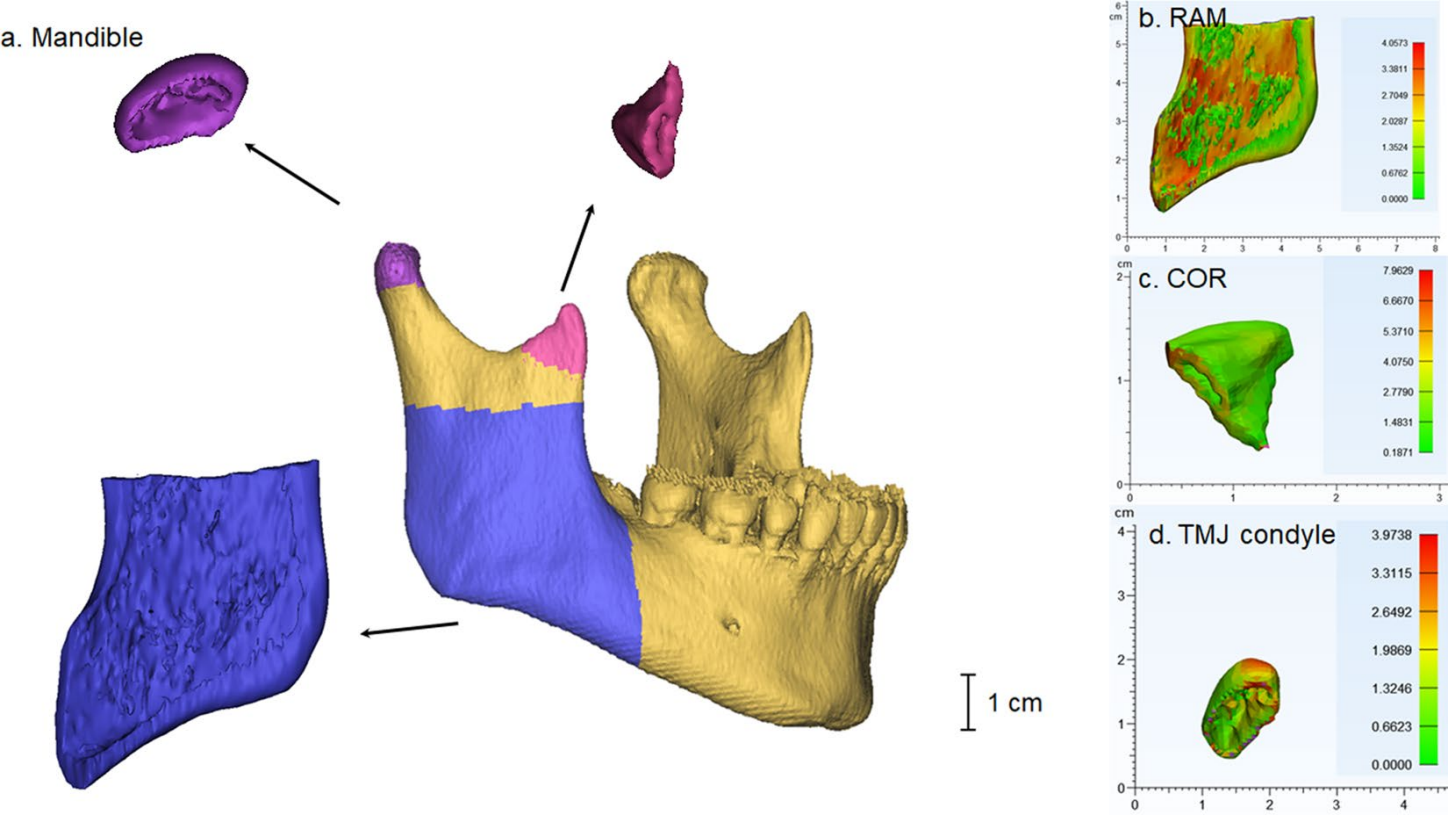
(**a**) 3D reconstructed mandibular model and separate sections. (**b**) Measuring average thickness of RAM by Mimics 22.0® software (**c**) Measuring average thickness of COR by Mimics 22.0® software (**d**) Measuring average thickness of TMJ condyle by Mimics 22.0® software.

CT data in the DICOM format were imported into Mimics 22.0® software (Materialise, Antwerp, Belgium) to reconstruct 3D models based on a standard HU thresholding method. The mask was created with voxels with the predefined HU. A previous report suggested that CT could facilitate the evaluation of the bone density by HU and that the HU of dense cortical bone in the mandible would be over 850 HU^43^ (Supplementary Figure). Therefore, the mandibular models were reconstructed using the masks with HU values over 850 (Fig. 3).

### Evaluation of the cortical bone density and cortical bone thickness

The tensile force developed by facial muscle contraction at its insertion sites and the compressive masticatory force transducing the load across the joints are two main force applied to the craniofacial area. We bilaterally calculated the cortical bone density and thickness of the insertion site of the temporalis and masseter muscles and two TMJ condyles where the masticatory forces were applied. The boundaries of the insertion site of temporalis and masseter muscles were determined on the mandibular mask; coronoid process of the mandible (COR) and inferior half of the lateral surface of the ascending ramus (RAM) of the mandible, respectively. The most prominent part of the condyle was defined as the boundary of the load bearing area (Fig. 3). The average HU and cortical thickness of the ROIs were automatically calculated using Mimics 22.0® software. To assess inter-examiner reliability, one orofacial pain and TMD specialist (JHK) and one orthopedic surgeon (SWH) evaluated 20 randomly selected CT data respectively and data from each examiner was compared (inter-examiner) using intraclass correlation coefficient (ICC) to assess the reliability. The ICC was 0.753 with statistical significance. One author (JHK) repeated the process after 1 week (intra-examiner) on 20 randomly selected CT data and data were compared using ICC and an acceptable agreement was detected. ICC was 0.857 with statistical significance.

### Statistical analysis

Power analysis indicated that a sample of 39 young (78 joints) and 38 post-menopausal females (76 joints) in a F test of one-way analysis of variance (ANOVA) with four groups would provide 83.5% and 82.3% statistical power at a 0.05 significance level with a large effect size (F = 0.40), respectively. Based on the Shapiro-Wilk normality test, our data were normally distributed; therefore, parametric tests were applied. To compare the differences of age, body mass index (BMI), muscle thickness, average HU and cortical thickness of the ROIs between young and post-menopausal females at an initial stage before intervention, an independent T-test was applied. Differences in the same variables among groups were compared separately in young and post-menopausal females with one-way ANOVA followed by post hoc analysis with Bonferroni’s test. Paired t-tests were used to assess changes in the same variables in accordance with the time change in each group. Differences in the degree of the orofacial pain, extents of CMO, MMO, and the number of TrPs among the four groups in both young and post-menopausal females were analyzed using three-way repeated measure ANOVA. All tests were two-sided and *P*-values less than 0.05 were considered statistically significant.

## Results

### Demographic characteristics of the patients

The age differences were not significant among the four groups in both young (*P* = 0.060) and post-menopausal females (*P* = 0.060). The BMI and duration of the orofacial pain also did not show statistical significance among four groups in both young and post-menopausal females (Table 1).

**Table 1:** Demographic features of patients in each group.

	CON (N = 6)	SP (N = 12)	BTXINJ (N = 11)	SPBTX (N = 10)	*P* value
**Young females**					
Age (years)	25.7 ± 5.5	26.9 ± 5.6	29.5 ± 7.5	31.0 ± 6.1	0.060
BMI	20.5 ± 1.7	20.4 ± 0.7	20.6 ± 1.9	21.4 ± 1.7	0.126
TMD Symptom duration (months)	50.0 ± 33.3	49.4 ± 41.5	30.8 ± 39.3	79.6 ± 104.5	0.102
	**CON (N = 6)**	**SP (N = 10)**	**BTXINJ (N = 12)**	**SPBTX (N = 10)**	***P*** **value**
**Post-menopausal females**					
Age (years)	54.5 ± 5.1	54.3 ± 4.5	58.4 ± 9.4	57.2 ± 9.2	0.060
BMI	20.6 ± 5.7	21.8 ± 2.6	23.3 ± 2.1	21.6 ± 2.1	0.120
TMD Symptom duration (months)	37.0 ± 41.3	36.9 ± 47.2	57.3 ± 69.0	31.0 ± 35.4	0.360

BMI, body mass index; TMD, temporomandibular disorder.

Descriptive values are shown as mean ± SD.

Data obtained from one-way ANOVA.

*P <0.05, **P <0.001 by one-way ANOVA.

### Clinical parameters related with TMD and the number of TrPs

The amount of CMO and MMO did not show statistical differences in the four groups in both young and post-menopausal females. The degree of the orofacial pain and the number of active and latent TrPs also showed no statistically significant differences among the groups in both young and post-menopausal females. However, all parameters showed symptomatic improvement after 12 months of intervention (T1) except for the number of latent TrPs. The duration of myofascial TMD also did not show statistical differences among the four groups in both young and post-menopausal females (Table 2).

**Table 2:** Degree of the orofacial myofascial pain among the four groups in young and post-menopausal females.

		CON		SP		BTXINJ		SPBTX		*P* value		
		T0	T1	T0	T1	T0	T1	T0	T1	Age difference	Intervention group	Time
CMO (mm)	Young	44.8 ± 4.1	47.5 ± 2.3	42.4 ± 5.5	47.4 ± 5.9	42.2 ± 3.8	47.5 ± 4.8	45.9 ± 4.7	51.4 ± 4.9	0.960	0.544	<0.001**
	Post-menopausal	40.5 ± 6.9	48.3 ± 5.2	42.5 ± 5.5	50.6 ± 3.5	43.5 ± 5.92	46.8 ± 4.0	41.8 ± 4.6	48.3 ± 5.2			
MMO (mm)	Young	46.5 ± 3.4	47.5 ± 2.3	45.0 ± 3.0	47.4 ± 5.9	44.3 ± 2.4	47.7 ± 4.9	47.7 ± 4.1	51.4 ± 4.9	0.737	0.837	<0.001**
	Post-menopausal	43.3 ± 6.0	48.3 ± 5.2	46.2 ± 1.2	50.6 ± 3.5	45.8 ± 4.7	43.8 ± 4.0	44.3 ± 4.4	48.3 ± 5.2			
VAS	Young	5.00 ± 2.10	3.17 ± 2.22	4.58 ± 1.83	0.92 ± 1.2	5.18 ± 1.99	1.64 ± 2.11	5.00 ± 2.54	1.70 ± 1.42	0.562	0.078	<0.001**
	Post-menopausal	5.00 ± 1.41	0.67 ± 0.82	3.80 ± 2.82	0.90 ± 1.60	5.08 ± 2.39	1.00 ± 0.95	6.60 ± 1.96	2.80 ± 2.70			
Number of active TrPs in masticatory muscles	Young	3.17 ± 0.98	1.67 ± 1.51	2.17 ± 1.53	1.67 ± 1.15	2.73 ± 1.68	0.73 ± 0.90	2.90 ± 1.20	0.60 ± 0.52	0.890	0.441	0.003*
	Post-menopausal	2.00 ± 1.10	1.50 ± 0.84	2.40 ± 1.43	1.80 ± 1.40	2.33 ± 1.07	0.83 ± 0.83	3.10 ± 0.99	1.40 ± 0.97			
Number of latent TrPs in masticatory muscles	Young	1.33 ± 1.51	1.67 ± 0.82	1.25 ± 0.87	0.67 ± 0.89	1.09 ± 0.83	1.64 ± 0.92	1.20 ± 0.92	0.80 ± 0.92	0.784	0.765	0.750
	Post-menopausal	1.67 ± 0.52	1.33 ± 1.03	1.10 ± 1.10	0.60 ± 1.26	1.08 ± 0.90	1.58 ± 1.44	2.30 ± 0.82	1.20 ± 0.79			

CMO, amounts of comfortable mouth opening without pain; MMO, amount of maximum mouth opening; VAS, visual analog scale; TrP, trigger point, T0, before treatment; T1, 12 months after treatment.

Descriptive values are shown as mean ± SD.

Data obtained from three-way repeated measure ANOVA.

*P <0.05, ** *P* <0.001 by three-way repeated measure ANOVA.

### Muscle thickness, cortical bone density, and cortical bone thickness in young and post-menopausal females before intervention

The thickness of temporalis and masseter muscles did not show significant differences among the four groups in both young and post-menopausal females at T0. Similarly, no significant differences in the average HU and cortical thickness of RAM, COR, and TMJ condyles among the four groups were observed in both young and post-menopausal females at T0 (Table 3).

**Table 3:** The thickness of masticatory muscles and density and thickness of the cortical bone of the ROIs in young and post-menopausal females before intervention (T0).

	CON (N = 6, 12 joints)	SP (N = 12, 24 joints)	BTXINJ (N = 11, 22 joints)	SPBTX (N = 10, 20 joints)	*P* value
**Young females**					
Temporalis muscle thickness (mm)	13.2 ± 2.6	13.2 ± 0.6	13.1 ± 0.9	13.9 ± 1.8	0.293
Masseter muscle thickness (mm)	14.0 ± 2.4	13.7 ± 1.6	13.3 ± 1.9	14.4 ± 1.3	0.235
Cortical density of RAM (HU)	1560.3 ± 15.9	1572.3 ± 28.6	1555.7 ± 34.6	1581.1 ± 43.1	0.076
Cortical thickness of RAM (mm)	1.59 ± 0.08	1.60 ± 0.11	1.56 ± 0.13	1.63 ± 0.11	0.239
Cortical density of COR (HU)	1475.0 ± 46.4	1484.5 ± 32.8	1463.0 ± 36.7	1487.5 ± 47.7	0.191
Cortical thickness of COR (mm)	1.88 ± 0.15	1.77 ± 0.19	1.76 ± 0.23	1.76 ± 0.11	0.219
Cortical density of condyle (HU)	1283.7 ± 58.3	1318.1 ± 43.7	1298.0 ± 32.6	1311.2 ± 44.4	0.122
Cortical thickness of condyle (mm)	0.79 ± 0.12	0.79 ± 0.08	0.80 ± 0.07	0.79 ± 0.07	0.940
	**CON (N = 6, 12 joints)**	**SP (N = 10, 20 joints)**	**BTXINJ (N = 12, 24 joints)**	**SPBTX (N = 10, 20 joints)**	***P*** **value**
**Post-menopausal females**					
Temporalis muscle thickness (mm)	11.1 ± 1.4	10.8 ± 0.8	11.2 ± 1.8	11.0 ± 1.3	0.845
Masseter muscle thickness (mm)	12.4 ± 1.0	12.2 ± 0.6	11.9 ± 0.6	12.0 ± 0.8	0.378
Cortical density of RAM (HU)	1438.6 ± 31.4	1439.2 ± 22.2	1428.5 ± 60.3	1451.3 ± 33.1	0.359
Cortical thickness of RAM (mm)	1.24 ± 0.22	1.22 ± 0.08	1.22 ± 0.15	1.30 ± 0.08	0.230
Cortical density of COR (HU)	1356.3 ± 30.7	1340.1 ± 21.1	1339.1 ± 29.9	1347.3 ± 38.6	0.237
Cortical thickness of COR (mm)	1.24 ± 0.18	1.30 ± 0.03	1.26 ± 0.09	1.32 ± 0.08	0.113
Cortical density of condyle (HU)	1277.6 ± 47.1	1235.5 ± 48.3	1223.6 ± 82.9	1257.4 ± 106.5	0.209
Cortical thickness of condyle (mm)	0.64 ± 0.16	0.64 ± 0.05	0.64 ± 0.04	0.64 ± 0.08	0.996

RAM, inferior half of the lateral surface of the ascending ramus; COR, coronoid process of the mandible; HU, Hounsfield unit.

Descriptive values are shown as mean ± SD.

Data obtained from one-way ANOVA.

* P <0.05, ** *P* <0.001 by one-way ANOVA.

The thickness of the temporalis (*P* < 0.001) and masseter muscles (*P* < 0.001) was significantly greater in young females compared to those in post-menopausal females before intervention (T0). The average HU of COR (*P* < 0.001) and RAM (*P* < 0.001) was also significantly higher in young females compared to those in post-menopausal females. The cortical thickness of the COR (*P* < 0.001) and RAM (*P* < 0.001) showed a similar tendency. The average HU (*P* < 0.001) and cortical thickness (*P* < 0.001) of the TMJ condyles were significantly higher in young females compared to post-menopausal females (Table 4). Thus, a lower thickness of the masticatory muscles and cortical bone quality of the muscle insertion site and load-bearing area were observed in post-menopausal females compared to those in young females.

**Table 4:** Age, body mass index, thickness of the masticatory muscles, and density and thickness of the cortical bone of ROI in young and post-menopausal females.

	Young (n = 39, 78 joints)	Post-menopausal (n = 38, 76 joints)	*P* value
Age (years)	26.9 ± 6.0	55.3 ± 6.3	<0.001**
BMI	20.7 ± 1.6	22.0 ± 3.2	0.003*
Temporalis muscle thickness (mm)	13.4 ± 1.5	10.8 ± 1.2	<0.001**
Masseter muscle thickness (mm)	13.8 ± 1.8	12.1 ± 0.8	<0.001**
Cortical density of RAM (HU)	1568.0 ± 34.2	1438.9 ± 41.7	<0.001**
Cortical thickness of RAM (mm)	1.59 ± 0.11	1.25 ± 0.13	<0.001**
Condylar cortical density (HU)	1305.4 ± 44.5	1244. ± 79.2	<0.001**
Condylar cortical thickness (mm)	0.79 ± 0.07	0.64 ± 0.81	<0.001**
Cortical density of COR (HU)	1477.7 ± 40.7	1347.3 ± 38.6	<0.001**
Cortical thickness of COR (mm)	1.78 ± 0.18	1.28 ± 0.10	<0.001**

BMI, body mass index; RAM, inferior half of the lateral surface of the ascending ramus; COR, coronoid process of the mandible; HU, Hounsfield unit.

Descriptive values are shown as mean ± SD.

Data obtained from independent t-test.

* P <0.05, ** *P* <0.001 by independent t-test.

### Effects of muscle intervention and masticatory loading on the cortical bone quality of the muscle insertion site

**Figure 4:**
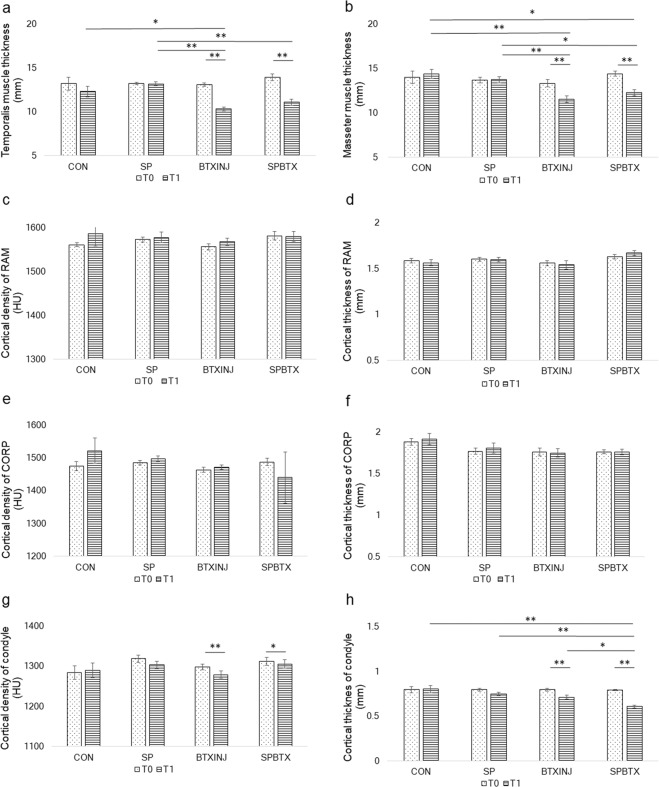
Changes of volume of masticatory muscles and cortical bone quality of the ROIs in young females between T0 and T1. (**a**) Temporalis muscle thickness (**b**) Masseter muscle thickness (**c**) Cortical density of RAM (**d**) Cortical thickness of RAM (**e**) Cortical density of COR (**f**) Cortical thickness of COR (**g**) Cortical density of condyle (**h**) Cortical thickness of condyle. Bars represented standard deviation of the mean. Differences of variables among groups were compared with one-way analysis of variance followed by post hoc analysis with Bonferroni’s test. Paired t-tests were used to evaluate changes in the variables in accordance with the time change in each group. *P < 0.05, **P < 0.001 by one-way ANOVA with Bonferroni’s post-hoc analysis and paired t-test.

**Figure 5:**
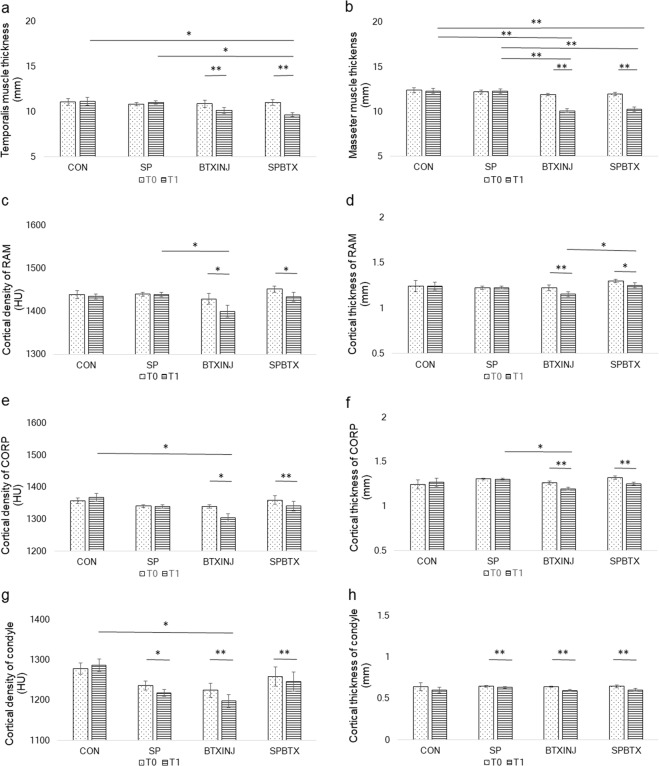
Changes of volume of masticatory muscles and cortical bone quality of the ROIs in post-menopausal females between T0 and T1. (**a**) Temporalis muscle thickness (**b**) Masseter muscle thickness (**c**) Cortical density of RAM (**d**) Cortical thickness of RAM (**e**) Cortical density of COR (**f**) Cortical thickness of COR (**g**) Cortical density of condyle (**h**) Cortical thickness of condyle. Bars represented standard deviation of the mean. Differences of variables among groups were compared with one-way analysis of variance followed by post hoc analysis with Bonferroni’s test. Paired t-tests were used to evaluate changes in the variables in accordance with the time change in each group. *P < 0.05, **P < 0.001 by one-way ANOVA with Bonferroni’s post-hoc analysis and paired t-test.

The thickness of the temporalis and masseter muscles was significantly decreased at T1 compared to T0 in BTXINJ and SPBTX in both young and post-menopausal females. The average HU and cortical thickness of RAM and COR did not show significant changes in young females between T0 and T1. On the other hand, the average HU values of RAM and COR showed significant decreases at T1 compared to T0 in BTXINJ and SPBTX in post-menopausal females. Average HU values of RAM and COR did not show significant changes in CON and SP in both young and post-menopausal females (Figs. 4 and 5). The quality of the cortical bone at the muscle insertion site were reduced after BTX injection in masticatory muscles and these effects were greater in post-menopausal females compared to those in young females.

### Effects of muscle intervention and masticatory loading on the cortical bone quality of load-bearing areas

In young females, the average HU of the condyle showed a significant decrease at T1 compared to those at T0 in BTXINJ (*P* < 0.001) and SPBTX (*P* = 0.014). The condylar cortical thickness was also significantly lower at T1 compared to T0 in BTXINJ (*P* < 0.001) and SPBTX (*P* < 0.001) in young females. In post-menopausal females, the influences of reduced masticatory loading and muscle thickness on cortical bone qualities seemed to be more prominent than those in young females. The average HU and cortical thickness of the TMJ condyle at T0 were significantly higher than at T1 in SP, BTXINJ, and SPBTX (Figs. 4 and 5). The cortical bone quality at TMJ condyles in both young and post-menopausal females were reduced after BTX injection in masticatory muscles and those influences were more remarkable in post-menopausal females.

## Discussion

The results from the present study demonstrated that the cortical bone qualities of the TMJ condyles, masticatory load bearing areas and COR and RAM, the masticatory muscle insertion sites seemed to be influenced by BTX injection in the temporalis and masseter muscle. Those effects were more prominent in the post-menopausal females compared to young females. The interactions between bone and muscle have become a topic of interest for both basic researchers and clinicians. The mechanical forces applied to the bone that originate from associated muscles are crucial to maintain skeletal health and bony integrity^3–5^. Animal studies which attempted to reveal the interactions between BTX-induced hindlimb muscle paralysis and atrophy may provide information about the effects of muscle contraction and unloading on maintaining bone structures^19,22,25^. This phenomenon also could be applied to the craniofacial area where reduced masticatory force after BTX injection may cause deterioration of the cortical and trabecular bony integrity of the condylar and alveolar bones^6,16–18,20,21,23,24^. Generally 0.2–1.0 U BTX injection in per masseter muscles in rodents^6,17,20,21,23,24^ and 10 U BTX injection in per masseter muscles in rabbits^16,18^ could elicit mandibular bone loss. Some human studies ever tried to investigate the effects of masticatory muscle paralysis induced by BTX injection on the mandibular bone quality. One study revealed that 30 U BTX injection in per masseter muscles and 20 U per temporalis muscles could cause reduced cortical bone thickness of the mandibular condyles and mandibular bone texture^28^. However specific protocols for dose and injection intervals of BTX injection which could elicit mandibular bony changes have not been established yet.

Decreased masticatory muscle thickness also seemed to have direct effects on bone density of the ramus and coronoid process of the mandible^16–18,23,24^. Most studies about the association between BTX induced masticatory muscle atrophy and mandibular bone loss have been animal studies and sparse well-designed human studies have been carried out due to difficulties in analyzing craniofacial bone qualities in humans. Owing to advances of 3D-modeling technology, evaluating the cortical bone density and the thickness of craniofacial structures using CT images has become possible in routine clinics. Therefore, in the present study, we aimed to reveal the influences of the BTX-induced masticatory muscle atrophy on the cortical bone thickness and density of the mandibular structures using CT imaging based on 3D modeling technology.

Aforementioned results showed that the thickness of the temporalis and masseter muscles and quality of the cortical bone of the mandible in post-menopausal females were significantly lower than those in young females. The aging process had a large impact on bone remodeling capacity and alteration of muscle-bone interactions. Elderly females with increased parathyroid hormonal levels and decreased estrogen levels could have a reduction in bone integrity over time^44,45^, and the associations among systemic osteoporosis, aging, and mandibular bone loss have been suggested^46–49^. Moreover, a generalized reduction in the total mass of the skeletal muscles and decreased size of the masticatory muscles in accordance with aging has been reported^50,51^. The masticatory function also seemed to be related to masticatory muscle thickness and systemic muscle mass^52,53^. Therefore, reduced cortical bone quality of the mandible in post-menopausal females could be influenced by systemic bone loss and decreased masticatory muscle thickness, with both having relevance with aging and the menopausal process.

Bone adapts to loading by altering its geometry and microarchitecture. Mechanical loading causes deformation of bones, and these strains activate mechanosensitive osteocytes that signal molecules to activate osteoblasts and osteocytes^54,55^. Aforementioned results showed that wearing a SS and having BTX injection in masticatory muscles elicited decreased cortical thickness and density of TMJ condyles, the masticatory load-bearing area. Similar previous results in rodents showed decreased cortical bone thickness after removal of gravitational loading of the hindlimbs as a result of decreased periosteal bone formation and increased endosteal bone resorption^25^. The effects of decreased masticatory loading on the cortical bone quality were more remarkable in post-menopausal females than in young females. Changes in sex hormone levels appeared to be the major cause, but the senescence of osteoblasts and of osteoprogenitor cells also played a role in the decreased bone remodeling capacity of the elderly^56^. The diminished ability to adapt to mechanical unloading and reloading in aged rodents has been suggested^41^ and our results which showed decreased adaptability of the condyles to decreased masticatory muscle thickness and functional loading especially in post-menopausal females could be understood in the same manner.

Therapy that included SS wearing and BTX-induced muscle inhibition had a combined effect on the cortical bone properties that were less than the total of individual effects of each intervention. This could be due to the dependent relationship between SS therapy and muscle force in patients with myofascial TMD. Several studies have mentioned that therapeutic mechanisms of SS therapy were related to factors involved in the modification of parafunctional activities and redistribution of overloading in the masticatory systems of patients with TMD and normal subjects^57,58^. BTX has been used to improve symptoms associated with TMDs and myofascial pain syndrome by reducing masticatory force and redistribution of masticatory loading. Because of the overlap in the therapeutic utility of BTX and SS in patients with TMD, the combined effect on the cortical bone properties would be less than the total of individual effects of each intervention. Therefore, as each intervention utilized in the present study could influence the other, the combined effect of BTX-induced muscle inhibition and SS wearing was less than the addition of each measure.

The results of the present study indicated that the effects of BTX induced muscle inhibition did not occurred only at the masticatory load bearing area. The cortical thickness and cortical bone density of RAM and COR showed significant decreases after BTX injection, especially in post-menopausal females. Previous animal models of rabbits showed similar results, with reduced cortical thickness at the masseteric ridge and the coronoid process after BTX injection in the temporalis and masseter muscles^17^. Therefore, reduced masticatory muscle thickness after BTX injection may lead to decreased cortical bone quality not only at the masticatory load bearing area but also muscle insertion sites. Interestingly, those effects seemed not to be prominent in young females. The diminished ability to adapt to mechanical unloading and reloading in aged rats has been observed^59^, however the influence of aged muscles at their attachment site remains obscure. Hence, we carefully suggest the possibilities that bones in post-menopausal females could have an altered adaptive response not only to mechanical unloading but also to reduced strain at the muscle insertion site.

3D imaging technology has been used for diagnosis and treatment planning in medicine and dentistry for decades, but sparse *in vivo* studies about bone properties using this technology have been undertaken. To the best of our knowledge, the present study is the first *in vivo* study that attempts to reveal the properties of cortical bone structures of the mandible using 3D-modeling technology. However, this study has several limitations. Firstly, because this study was based at a tertiary medical center, it might provide limited information about the interactions among cortical bone quality and muscle thickness in ordinary individuals. Because all participants in the present study were patients with myofascial TMD, the degree of orofacial pain could influence the masticatory performance. Secondly, due to a lack of data about systemic bone mineral density, the results could show knowledge about the associations between muscle and bone in a restricted focal area. Thirdly, the present study only provided information about the cortical bone quality of the mandible not trabecular bone quality. Despite of advances of 3D modeling technologies, no reliable and valid method for evaluating trabecular bone qualities using conventional CT data has been developed. Finally, because of the characteristics of the retrospective study, the present study inevitably had problems with bias in sample selections. However, to overcome this limitation, homogeneity of the samples in each group including initial status of the orofacial pain and the cortical bone quality was confirmed through statistical methods. Future prospective community-based cohort studies including systemic bone mineral density data and focal trabecular bone quality data of the mandible with ordinary individuals in diverse age ranges would be necessary to elucidate the role of masticatory muscles in bone quality of the craniofacial structures.

The results from the present study demonstrated that BTX-induced masticatory muscle atrophy could alter the mandibular cortical bone quality in both young and post-menopausal females. The effects of decreased masticatory muscle thickness on the cortical bone quality of muscle insertion sites seemed to be more prominent in post-menopausal females compared to young females. Reduced masticatory muscle thickness could alter the condylar cortical bone structure in both young and post-menopausal females. Therefore, comprehensive and integrated understanding of masticatory function, the muscle strain to bone, and cortical bone quality is necessary to preserve bone health in the craniofacial area.

## Supplementary information

Supplementary Figure.l.

## Data Availability

The datasets used and/or analyzed during the current study are available from the corresponding author on reasonable request.
