# Supplementary material for: Decreased mandibular cortical bone quality after botulinum toxin injections in masticatory muscles in female adults
Source: Sci Rep. 2020 Feb 27;10:3623. doi: 10.1038/s41598-020-60554-w (PMC7046747; doi:10.1038/s41598-020-60554-w)
Supplement: Supplementary file 1 — Supplementary Figure.l. [file 41598_2020_60554_MOESM1_ESM.docx]

**Decreased mandibular cortical bone quality after botulinum toxin injections in masticatory muscles in female adults**

**Authors:**

^1^Seok Woo Hong, MD., ^2*^Jeong-Hyun Kang. DDS, PhD.

^1^Department of Orthopedic Surgery, Kangbuk Samsung Hospital, Sungkyunkwan University School of Medicine, 29, Saemunan-ro, Jongno-gu, Seoul, 03181, Korea (ROK) (ORCID: 0000-0003-4059-1557)

^2^Clinic of Oral Medicine and Orofacial Pain, Institute of Oral Health Science, Ajou University School of Medicine, 164, Worldcup-ro, Yeongtong-gu , Suwon, Gyeonggi-do, 16499, Korea (ROK) (ORCID: 0000-0001-7124-8693)

***Corresponding author:**

Jeong-Hyun Kang, DDS, PhD

Clinic of Oral Medicine and Orofacial Pain

Institute of Oral Health Science

Ajou University School of Medicine

164, Worldcup-ro, Yeongtong-gu

Suwon, Gyeonggi-do, 16499

Korea (ROK)

Tel: +82-31-219-7456

E-mail: [irene85@snu.ac.kr](mailto:irene85@snu.ac.kr)

**Date of re-submission:** 7th, February, 2020.


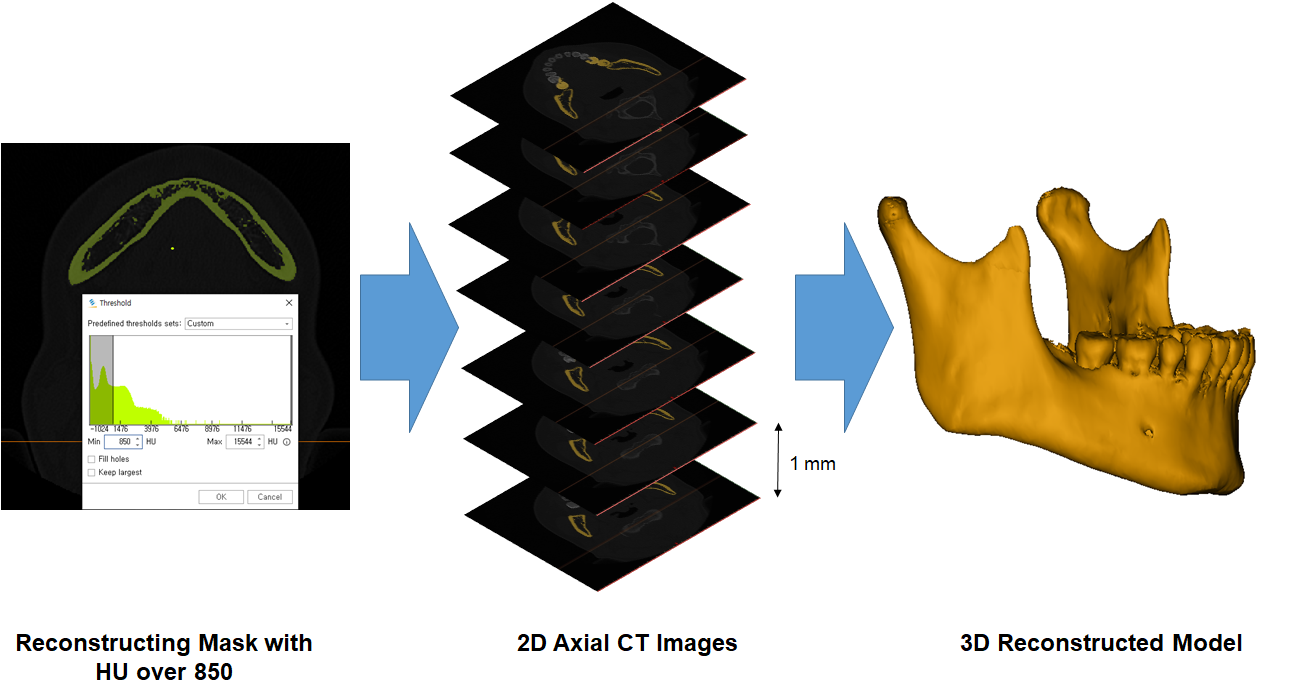


**Supplementary Figure 1.** Workflow of reconstructing 3D mandibular model
